# MOST+: A de novo motif finding approach combining genomic sequence and heterogeneous genome-wide signatures

**DOI:** 10.1186/1471-2164-16-S7-S13

**Published:** 2015-06-11

**Authors:** Yizhe Zhang, Yupeng He, Guangyong Zheng, Chaochun Wei

**Affiliations:** 1 School of Life Sciences and Biotechnology, Shanghai Jiao Tong University, 800 Dongchuan Road, Shanghai 200240, China; 2 Shanghai Center for Bioinformation Technology, 1278 Keyuan Road, Pudong District, Shanghai, 201203, China; 3 Bioinformatics and Systems Biology Program, University of California, San Diego, 9500 Gilman Drive, La Jolla, CA 92093, USA; 4 CAS-MPG Partner Institute for Computational Biology, Shanghai Institutes for Biological Sciences, Chinese Academy of Sciences, 320 Yueyang Road, Shanghai 200031, China; 5 Program in Computational Biology and Bioinformatics, Duke University, Durham, NC 27708, USA

**Keywords:** *De novo *Motif finding, Epigenetics, ChIP-seq, Suffix tree, DNase I hypersensitivity sites

## Abstract

**Background:**

Motifs are regulatory elements that will activate or inhibit the expression of related genes when proteins (such as transcription factors, TFs) bind to them. Therefore, motif finding is important to understand the mechanisms of gene regulation. *De novo *discovery of regulatory elements, like transcription factor binding sites (TFBSs), has long been a major challenge to gain insight on mechanisms of gene regulation. Recent advances in experimental profiling of genome-wide signals such as histone modifications and DNase I hypersensitivity sites allow scientists to develop better computational methods to enhance motif discovery. However, existing methods for motif finding suffer from high false positive rates and slow speed, and it's difficult to evaluate the performance of these methods systematically.

**Result:**

Here we present MOST+, a motif finder integrating genomic sequences and genome-wide signals such as intensity and shape features from histone modification marks and DNase I hypersensitivity sites, to improve the prediction accuracy. MOST+ can detect motifs from a large input sequence of about 100 Mbs within a few minutes. Systematic comparison method has been established and MOST+ has been compared with existing methods.

**Conclusion:**

MOST+ is a fast and accurate *de novo *method for motif finding by integrating genomic sequence and experimental signals as clues.

## Background

Gene expression can be activated or inhibited when proteins (transcription factors, TFs) bind to a genomic sequence segment (i.e. motif) dispersed in promoter and enhancer regions. To gain insights into the mechanisms of gene regulation, the patterns and locations of functional TF binding sites should be identified. Nevertheless, they remain to be challenging problems until high-throughput approaches like ChIP-seq (chromatin immunoprecipitation followed by massively parallel sequencing) [1,2] and ChIP-chip (ChIP on chip) appeared. However, ChIP-seq methods have poor resolution ranging from 200 bps to 300 bps. Besides, their results depended on high quality antibodies for the TFs, which are hard to obtain in general. Moreover, despite the possibility of examining multiple TFs in a single experiment [3,4], it is still prohibitively expensive to test thousands of TFs in various cell types and conditions. Therefore, computational methods are still in a great demand as complementary means to analyze TF binding sites, like PWM (Position Weighted Matrix) guided TFBS identifying and motif discovery.

The major task of motif discovery (or "motif finding") could be viewed as deciphering hidden patterns significantly over-represented in a given genome. Alternative binding motifs, spacing in motifs, palindromes, tandems of single motifs and binding via cofactors make this task complicated. Besides, some cis-regulatory elements are highly degenerated or involve complicated dependency among positions, which is presumably derived from biochemical structure [1], rendering it difficult to discover them. As a result, a motif found by existing algorithms is an approximation instead of an exact reflection of functional binding patterns.

Two types of motif identifying approaches were developed to detect motifs: one uses orthologous sequence alignments extracted from multiple genomes, and the other uses genome regions enriched with particular motifs in a single genome [3]. The first type of methods, such as MEME [5], NestedMICA [6], and Gibbs sampler [7] utilize Bayesian model, EM algorithm, or Gibbs sampling techniques to obtain a Maximum A Posteriori (MAP) for motifs with a given length. Based on these methods, they iteratively update the motif and position with an expectation step followed by a maximization step until some termination criteria are reached [8]. These settings fit quite well with problems of extracting shared sequences from multiple genomes or a small subset of promoter regions (or enhancer regions believed to share some motifs). However, the majority of these methods are slow when processing big datasets. For example, it takes MEME [9] a few days to deal with input sequences of 0.5 Mbps. In addition, due to the probabilistic nature of the models, they are more or less sensitive to noises in input data. Efforts have been made to improve these classic methods over the last few years. STEME [10] speeds up MEME by indexing sequences with a suffix tree. ChIPMunk [11] searches a gapless multiple local alignment using a greedy algorithm to deal with a large set of inputs.

On the other hand, the other type of approaches, word-frequency-based approaches, have been developed, such as WEEDER [12], MDScan [13], Trawler [14], Amadeus [15], DREME [16], and CisFinder [17]. These tools demonstrate significant reduction in computational time and are often better devised for single genome analysis with a large input. Typically, they exhaustively enumerate all possible arrangements of all nucleotides up to a user-specified length and select those words (k-mers containing no degenerated nucleotide) that occur significantly more frequent than those in the Markov background. Some methods iterated by masking stronger motifs first and to find weaker motifs later, while others try to identify all significant motifs, which will then be refined (or clustered) elaborately by expectation-maximization, Self Organizing Map (SOM) [18] or hierarchical clustering. Suffix tree, a data structure to represent the organization of all suffixes of a string, has often been utilized to index input sequences and speed up sequence searching in some word-count based methods like Trawler [14], STEME [10], WEEDER (v1.4.2) and DRIMUST [19]. With the Ukkonen algorithm, the whole process can be completed in linear time in terms of the size of input [10]. One major challenge for typical word-count methods is that motifs with weak patterns may be discarded and many spurious motifs may be generated. As a result, a motif can barely be differentiated from the background if its occurrence is relatively low. Limitations like this can hardly be eliminated especially when the size of the input sequences is small [17].

We hope to enhance the word-count methods by utilizing genome-wide signatures from experimental data. Many *in vivo *events (iVEs) [20], such as open chromatin accessibility or epigenetic modification, strongly associated with the TF binding process, indicating their potential use as biological context to identify TFBSs. For instance, with genome-wide nucleosome occupancy profiles (identified by DNase-Seq or FAIRE-Seq [4,21] in a same cell line, the search for TFBSs on genomic sequence could be confined to only regions with open chromatin accessibility. Meanwhile, epigenetic modifications, especially several kinds of methylations on histones, have also been proved very informative for TFBS finding [4,21]. These modifications can either be associated with activation or repression of corresponding genes in different cell types [22].

Some recent algorithms have already taken advantage of histone mark information to identify individual occurrences of known motifs, such as CENTIPEDE [4], CHROMIA [23], FIMO [21] and HINT [24]. Considering a large collection of TFBSs and other CRMs (cis-regulatory modules) are still unrevealed, and sources like histone modification signals can be informative, there is some space to develop better motif finding approaches by taking advantage of epigenetic profiles around TFBS instances.

In this paper, we present MOST+, which can *de novo *predict motifs and their loci in a genome with the guidance of histone modification and DNase I hypersensitivity information. MOST+ first finds overrepresented words using a suffix tree, then incorporates external genome-wide signals to enhance the search for significant seeds and the aggregation of seeds into motifs. We show that our algorithm integrates successfully the intensity and shape features of external signals to promote the accuracy of motif finding.

## RESULTS

**Figure 1:**
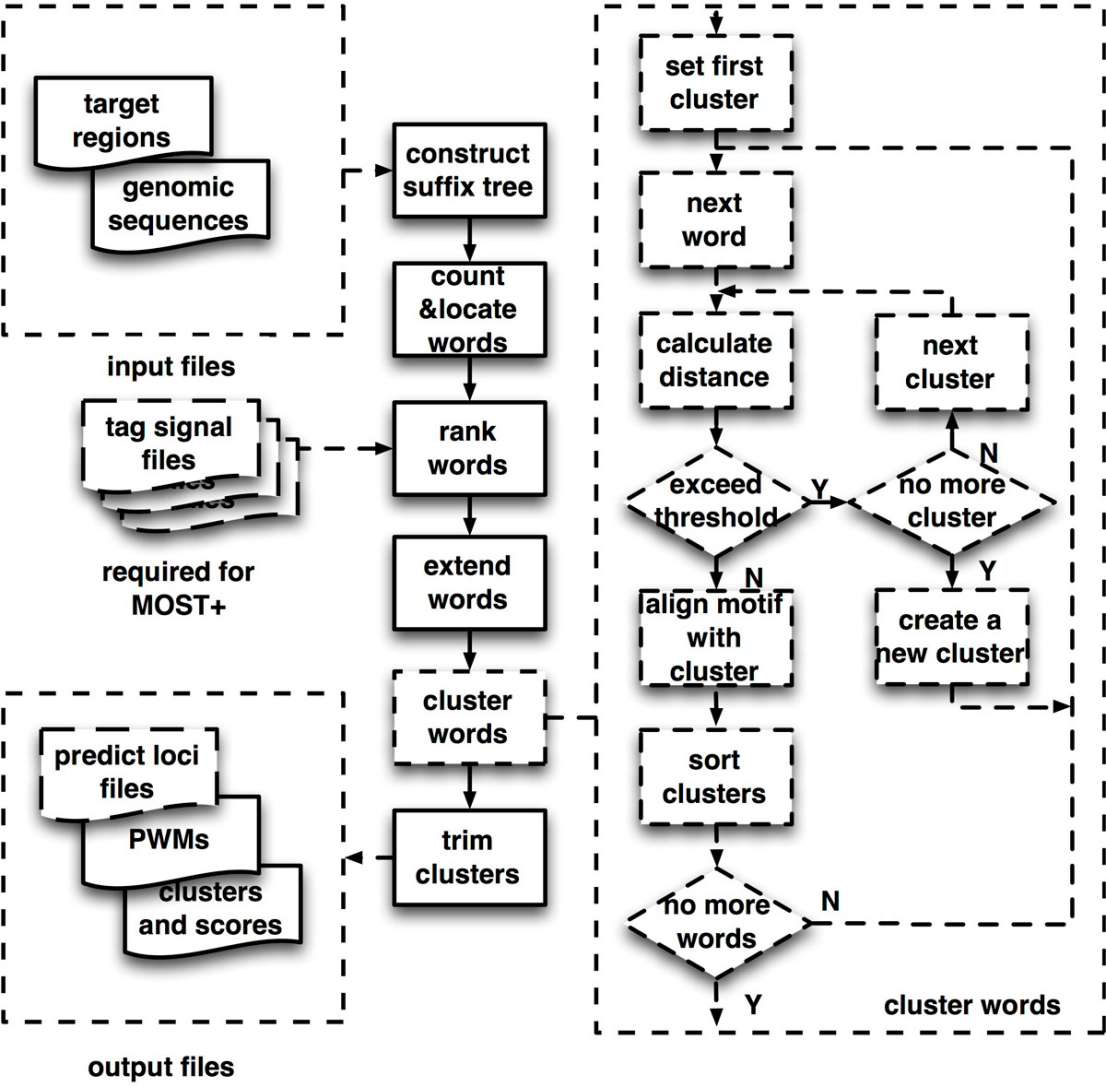
**The pipeline of MOST+ system**. A set of target genomic sequences are extracted from a genome then indexed by a suffix tree to count occurrence of each word (or K-mer). If under MOST+ mode, histone modification marks and/or DNase I hypersensitivity (referred as tag signals in this schema or mark distribution) of each word are used to yield mark distribution scores. Top ranked words are put into clustering and motifs are generated from the resulted clusters. The strategy for clustering is illustrated on the right panel of this figure.

We have created fast motif finding methods MOST and MOST+. The system diagram of MOST/MOST+ was shown in Figure 1 and described in Methods section. MOST uses only sequences as the inputs while MOST+ can integrate additional experimental data.

### Comparison of MOST+/MOST with existing algorithms

MOST+ and MOST were compared with several prevalent motif finding systems, such as MEME, Trawler, WEEDER, CisFinder and HOMER, in terms of speed and accuracy. Different fragments of the mESC ChIP-seq dataset and the genome-wide promoter regions of the mouse genome were used as the gold standard datasets (see Methods for more details).

### Comparison of speed

**Figure 2:**
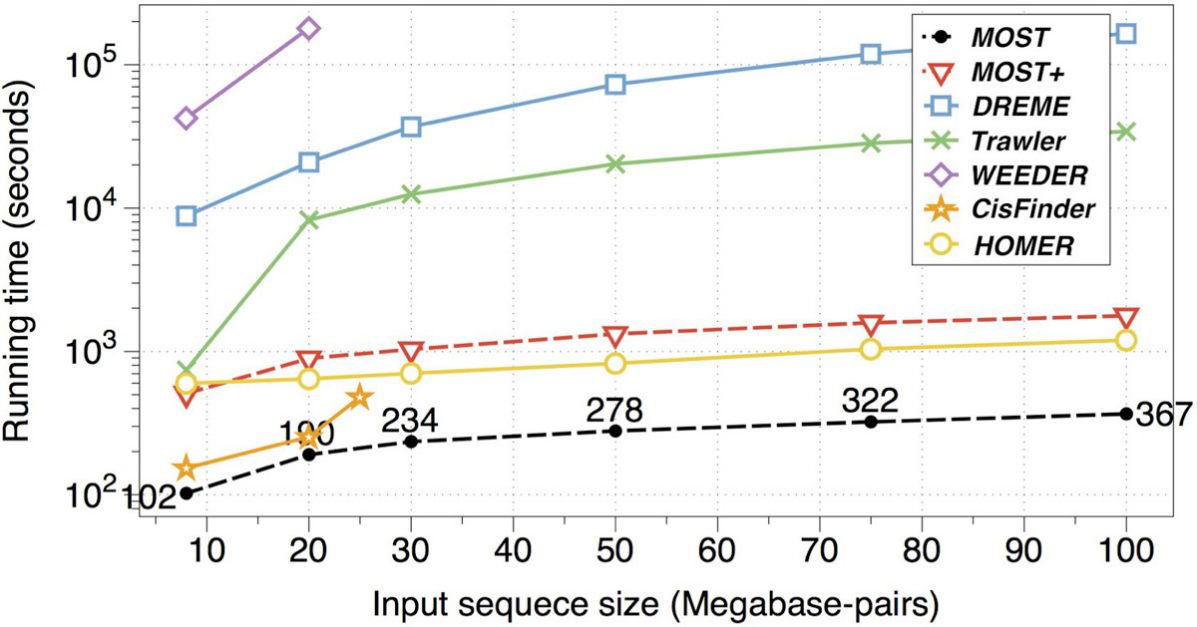
**Comparison of different motif finding methods**. X-axis is the running time in logarithmic scale while the Y-axis is the total size (Mbps) of input sequences.

MOST outperformed all tested algorithms (Figure 2, Table S1) in terms of speed for all datasets with various sizes. MEME [9] had not produced any result after 4 days running on a 16 Mbps dataset. WEEDER failed to produce a result when the input sequence length exceeds 20 Mbps. CisFinder was fast, but had a restriction on the input size (up to 25 Mbps). In short, MOST+/MOST and HOMER [25] (version 4.2) were able to produce results for large datasets in a reasonable time.

For all methods, default parameter settings were used when possible (details can be found in Additional file 1). Word lengths were chosen to a range from 4 bps-7 bps, with an exception of WEEDER (up to 8 bps).

MOST+ is insensitive to the input size. Counting and locating words takes little time. Memory consumption remains relatively low when data size grows up to 100 Mbps (linear spatial complexity, MOST+ requires 500 megabyte memory for an 8 Mbps input).

### MOST/MOST+ can find complicate motif patterns

MOST/MOST+ was designed to bridge gaps and find long gap patterns. For instance, Sox2 (canonically represented by CATTGTT) and Oct4 (or known as POU5F1, canonically represented by ATGCAAAT) often occur as a heterodimer (characterized by motifs of OCT-N-SOX or OCT-3N-SOX). These motifs had been detected simultaneously (Figure S1).

MOST+ also successfully detected motif of SMAD1, which has relatively low occurrences in tested ChIP-seq peak regions. For OCT4, MOST+ successfully detected a tandem repeat of OCT4 core motif (Figure S1).

Alternative TFBSs and palindromes were found for ESrrb (Estrogen-related receptor beta) when a larger word width (K = 11) was employed to capture more sophisticated co-occurrence and a more stringent clustering threshold was set to discriminate sub-patterns (Figure S1).

### Incorporating epigenetic marks helps filter out non-functional motifs

MOST reported a similar result compared to the prevalent algorithms. After epigenetic signals were added (MOST+ mode), motifs were better aligned with known motifs in databases and the rank of essayed TF was lifted (Table 1).

**Table 1:** The impact of genome-wide signals on prediction accuracy (MOST vs. MOST+, K=9)

TF	ChIP-seq Peaks	Ranking	Predicted sites	Co-factors
CTCF	39,609	1→1	27,458→43,150	0→2
ESRRB	21,647	1→1	17,144→18,998	3→4
KLF4	10,875	1→1	7,662→10,900	5→8
OCT4	3,761	1→1	2,051→2,802	3→7
cMyc	3,422	2→1	1,120→1,342	3→6
nMyc	7,182	2→1	1,853→2,519	3→4
SMAD1	1,126	9→4	119→135	4→8
E2F1	20,699	-	-	3→5
NANOG	10,343	2→1	2,844→3,012	2→3
SOX2	4,526	1→1	3,515→3,490	3→6
STAT3	2,546	1→1	1,486→1,560	4→7
TCFCP2L1	26,910	1→1	1,4568→1,4780	3→7
ZFX	10,338	1→1	4,269→7,684	3→4

Column 2-5: 2) the number of ChIP-seq peaks; 3) the change of ranks for the major TFBSs (from MOST to MOST+); 4) number of predicted binding sites for each TF; 5) the change of numbers of co-factors predicted by MOST and MOST+.

**Figure 3:**
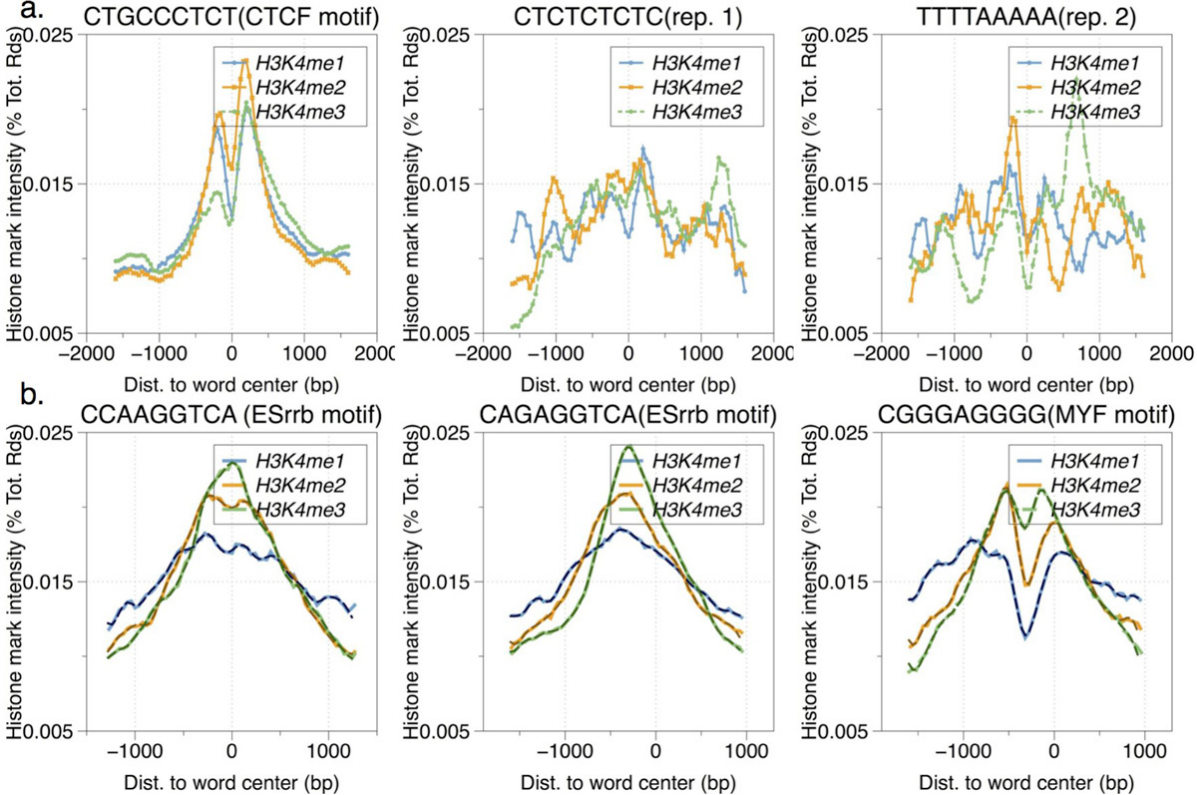
**Distributions of several highly enriched word instances found in CTCF and ESrrb's ChIP-seq dataset: (A)**. The upper 3 figures are from CTCF dataset. Spurious words show irregular or flat patterns (CTCF word "CTGCCCTCT" versus repeat words: "CTCTCTCTC", "TTTTAAAAA". All three words have odds ratio scores ranging from 3.4 to 4, i.e. in the same level of over-representative ratio), indicating one can make use of tag signals to discriminate motif words from their background. (B). The lower 3 figures are from ESrrb dataset. Distributions of word from Esrrb motif ("CCAAGGTCA" and "CAGAGGTCA", both contains core 'AGGTCA') strongly resemble to each other, while MYF motif component word (lower right corner: "CGGGAGGGG") shows a distinct pattern in distribution (dotted lines show distributions smoothed by a DFT with the top 5/8 higher frequency components removed).

We observed that spuriously over-represented k-mers (like some tandem repeat that may not be motif in our dataset) were more likely associated with a higher level of noise and asymmetry (Figure 3a, and Figure S2). Indicating these features may help eliminating false positives. Furthermore, similar motifs can have distinct mark distribution patterns (Figure 3b). If guided by epigenetic marks, it would be possible to distinguish similar motifs.

In general, MOST+ found more motifs that exist in motif database than MOST, which means more essayed TF or co-factors are found. According to Bieda et al.,[26] the motif of E2f1 is very hard to be identified maybe due to indirect binding. However, MOST+ did report a motif (featured by CGCCAT) that ranked second and resembled the motif of E2f-family member E2f3. Detections of n-Myc, c-Myc, zfx and SMAD1 would also benefit from epigenetic marks in terms of highlighting the assayed motifs.

### Comparison of motif accuracy on ChIP-seq datasets for mouse and human

MOST+ and exiting motif-finding tools were compared using the ChIP-seq dataset of 13 TFs of mouse ES cell.

General comparison was conducted based on alignments with known motifs in the reference database. Comparison on other 5 algorithms showed that MOST+ was among the best algorithms in both capturing major TFBSs (the binding sites of the assayed TFs) and detecting co-factors. MOST+ and HOMER identified 11 of 13 major motifs (with the major motif ranked the first in results) with significant e-value of alignment whereas DREME reported 10 motifs (Table 2). MOST+ can recover a comparable amount of co-factors with DREME, which was devised for finding co-factors (Table 3). Like CisFinder, MOST+ could automatically determine a self-adaptive length for each cluster.

**Table 2:** Accuracy comparison with existing methods

Algorithms	Detection ratio	Succeeded	Co-factors	Motif cluster
MOST	43%	8	3.7	Y
MOST+	45%	11	4.3	Y
DREME	25%	10	5.6	N
Trawler	11%	8	0.6	N
nestedMICA	21%	10	2.1	N
MEME	5%	10	0.9	N
WEEDER	6%	10	0.5	N
CisFinder	76%	10	3.6	Y
HOMER	38%	11	3.0	Y

Columns 2-5: 2) Detection ratio: the number of clusters aligned to motifs in the database divided by the total cluster number found over 13 TFs; 3) Succeeded: numbers of major TFBSs ranked first in the results; 4) Co-factors: the average numbers of unique co-factors found in databases (e-value<0.05 given by TOMTOM) and 5) Motif cluster: whether a clustering step is used to merge results of a method.

**Table 3:** Co-factors found by MOST+

TF	Co-factors uniquely found by MOST+	Co-factors both found by DREME and MOST+	Co-factors uniquely found by DREME
CTCF	E2F3^a^, Myf		Myc, GABPA, STAT
ESRRB	Myf^a^, Sp1^a^, Srf	Klf4^a^	STAT3, Oct4, Myc, Sox2
KLF4	FEV^a^, CREB, CTCF^a^, Egr1^a^	Esrrb, STAT3, sp1^a^, Sox2, Oct4	Oct4, Gata3,Myc, Zfp161^a^
Oct4	GABPA^a^, Zfx^a^, CTCF^a^, Stat3, sp1^a^	Sox2^a^, Esrrb^a^, Klf4^a^	CREB/ATF
cMyc	CREB^a^, GABPA^a^, Klf4^a^, Sox2, YY1^a^	STAT3^a^	Egr1^a^
nMyc	Elf3^a^, GABPA^a^, Zfp161^a^,	CREB/ATF^a^	STAT3, Smad1, sfpid
SMAD1	Sp1^a^, Sox11^a^, REST, FEV,Spib	Sox2^a^, Oct4^a^, Klf4^a^,Esrrb^a^	Zic3^a^, Zfp740^a^
E2F1	Sp1^a^, Myf, GABPA^a^	STAT3^a^, CREB/ATF	Myc^a^, FOX
NANOG		Zic3^a^, Klf4^a^, Esrrb^a^	Elf5^a^, Tead1
SOX2	Sox10^a^, CTCF^a^, Myf^a^, Runx1^a^	Oct4^a^, Klf4^a^, Esrrb^a^	Zic3^a^, STAT3
STAT3	Zic3^a^, Jundm2, FEV^a^	Esrrb^a^, Klf4^a^, Oct4, Sox2, sp1^a^	Myc, Irf4
TCFCP2L1	Sox4^a^, Zic3^a^, Myf	Klf4^a^, Esrrb^a^, Sox2,Oct4, Sp1^a^	Egr1^a^, Fox^a^, Myc, Tead1, CREB/ATF, STAT3
ZFX	Myf^a^, Egr1^a^, sp1^a^	STAT3	Myc^a^, Esrrb

^a ^Supported by CisFinder or HOMER

Columns 2-4: Co-factors found by 2) MOST+ only; 3) both MOST+ and DREME; and 4) DREME only for each dataset. When more than one motifs in the database were aligned, only the one with lowest p-value was counted.

**Figure 4:**
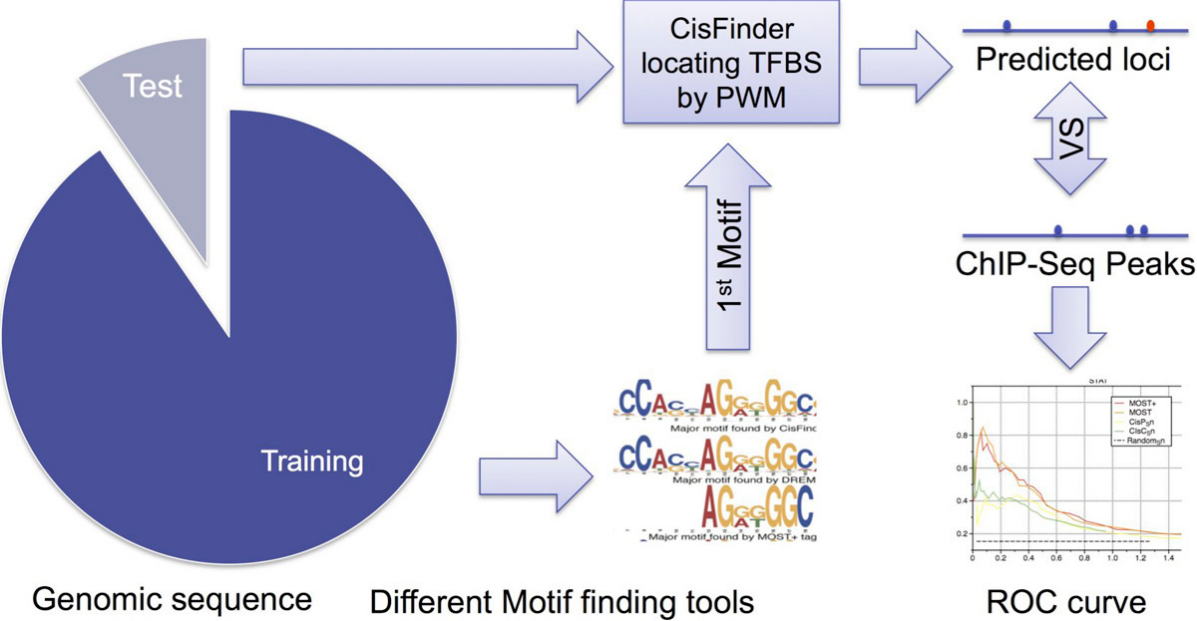
**The diagram of pipeline for parameter optimization and method comparison**. A motif-finding step is followed by a TFBS identification step (by CisFinder) using motifs and genomic sequences as input. Training data (8 of 10 folds) are fed into motif finding tools, and then accuracy is evaluated based on how well the motifs recovered can pinpoint TFBSs. AUROC is used to represent the accuracy of each method.

**Figure 5:**
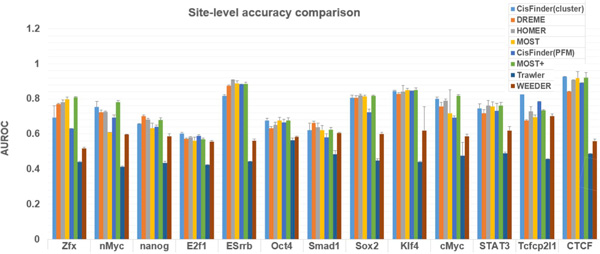
**Comparison of site-level accuracy for different methods**. AUROC of each method on recovering motifs for different essayed TFs were shown in the figure.

Assessment on site-level accuracy of motif finding methods was conducted by using the pipeline described in Methods section (Figure 4). Figure S3 showed a comparison of found motifs for different methods. Results show MOST+ has the highest AUROC sum over 13 TFs, though the situation varies from TF to TF (Figure 5, Figure S4). With parameters learned by part of the dataset, MOST+ achieved better accuracy on recovering motif positions with validation data, suggesting that motifs could reflect actual binding sites better if external signals were available. The AUC (Area Under the Curve) of ROC increased when word counts and mark distribution scores were combined under the appropriate parameter setting. This supported the idea that epigenetic marks can be helpful to cluster words [4].

With this parameter setting learned from training and validation datasets, we compared MOST+ with other 5 algorithms on the remaining partition data. The learned parameters were given in Table S2 (see Additional file 1 for more detail). Each time, we included one feature in our model to test its contribution to the accuracy improvement. Results show that the mere use of asymmetry feature contributes the most to the overall improvement of AUROC, while the utilization of original signals of essayed TF ChIP-seq data failed to show any advantage.

**Figure 6:**
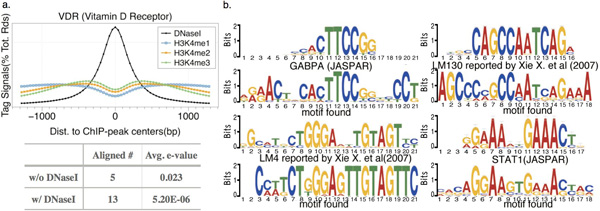
**(A)**. DNase I hypersensitivity signal shows evident cleavage pattern around ChIP-seq peaks in human LCL datasets. (B). With the help of DNase I hypersensitivity signals, additional motifs were found by MOST+ in VDR datasets). Some long motifs are similar to those reported in Xie et al. (2005,2007) [2]36.

To validate the power of our algorithm, we also applied the model to the human data. On average, MOST+ outperforms MOST by better AUROC, however, with an exception of JUND 1 and 2 (Figure S5). When epigenetic signals were added to the model, MOST+ found some novel co-factors that MOST could not find. For instance, with the aid of DNase I hypersensitivity information, additional motifs that strongly resembled STAT1, GABPA, LM4 and LM130 (both were long motifs reported by Xie et al.) were found in VDR (Vitamin D Receptor) datasets (Figure 6).

### Genome-wide promoter analysis

To date, large portions of TFBSs are still undetected. However, poor performance has often been reported on capturing motifs in generic promoters [27]. With the power of epigenetic marks, we hoped our overall finding of motifs in all regions close to the transcription start sites (TSSs) might reveal novel motifs. We took an exhaustive search on 89 Mbps mouse promoter regions. When more hints from ChIP-seq data were available, we put on a more stringent threshold of word clustering and masked repeats (see Additional file 1 for MOST+ command lines).

**Figure 7:**
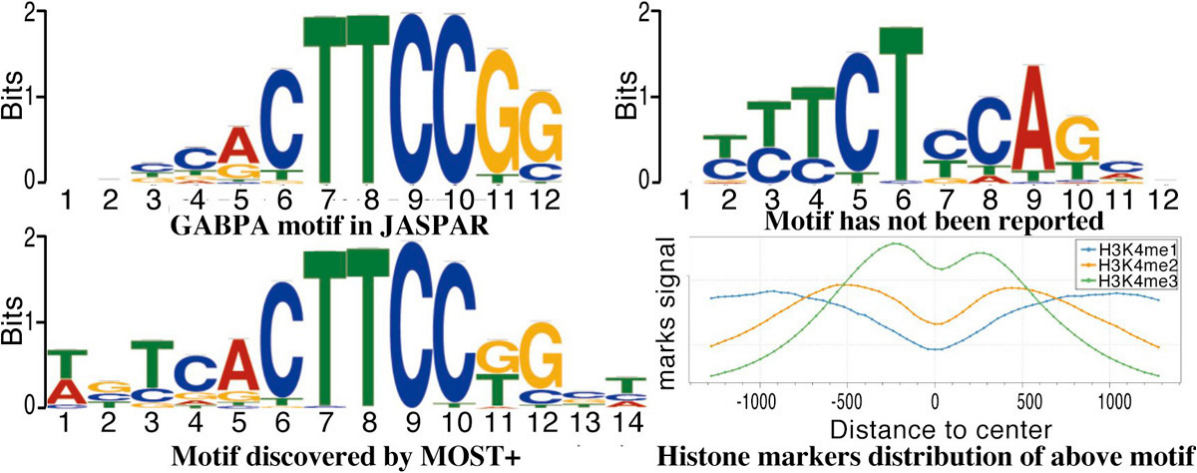
**Motifs discovered by MOST+ in all promoters of mouse genome**. Left panel: A motif discovered by MOST+ that resembles GABPA motif in JASPAR. Right panel: Examples of some unknown motifs with obvious kurtosis pattern in histone modification distributions.

Results showed that 117 motifs discovered by MOST+ (16% of total found motifs) could be aligned with one or more motifs in our mouse motif databases (JASPAR, UNIPROBE and Chen et al., 541 in total, Figure S6). Left panel of Figure 7 showed an example of motifs discovered by MOST+. MOST+ also output hundreds of novel motif candidates, which were not found in mouse motif databases. However, the mark distributions centred by some of these motifs show non-trivial shapes. This indicates either a putative novel motif or a potential sequence mark of epigenetic modifications (right panel of Figure 7).

## DISCUSSION

### 

Our results demonstrated that chromatin accessibility and epigenetic information should not be underestimated for their potential to identify motifs and their co-factors. In the near future, it is possible to utilize these marks better by introducing different model frameworks, such as conditional random field (CRF [28]) framework, which can estimate the weight for each feature automatically and predict the binding loci more precisely.

This algorithm can be improved in many ways. A parameter advisory phase can be included before MOST+ is executed. More sophisticated analysis like wavelet analysis may be employed to extract oscillation features of mark distributions. The improvement on resolution of ChIP-seq will make the distribution more informative, which can potentially boost the accuracy of the algorithm. Some aspects of the algorithm improvement are discussed as follows.

#### Setting parameters for epigenetic signals

Parameters for different epigenetic signals and different genomes vary. To make the most out of external signals, the optimal parameters should be learned from the data. However, different genomes may share some common epigenetic features. Users are encouraged to use our pre-set parameters for their genomes of interest with signals like DNase I, H3k4me1 and H3K4me3. Although H3K4me2 is informative in human datasets, it was not informative in mouse dataset (result not shown). Therefore, it was not included as the default signals.

#### Impact of the window size K

Since the results and running time of MOST+ depend on the window size K, we examined its performances on detecting major motifs of CTCF with distinct K values. Often but not necessarily, bigger K values could capture sophisticated patterns like gaps or alternative binding motifs. However, a very large K could be neither spatially nor temporally efficient in terms of computing. Therefore, we set K = 9 as default for typical ChIP-seq data (with 100 bps peaks) to quickly depict a whole landscape of a certain motif without losing much sensitivity (see Table 4 for details).

**Table 4:** Impact of parameter K on MOST/MOST+.

Mode	K	Time(s)	Motifs predicted	E-value
MOST	7	110.34	2	0.09
	8	111.85	5	4.87e-12
	9	133.06	5	6.35e-21
	10	448.22	5	1.02e-22
	11	2786.11	6	4.78e-20

MOST+	7	481	5	3.03e-05
	8	510	8	3.95e-13
	9	527	12	3.77e-21
	10	753	14	7.50e-27
	11	3244	6	1.07e-18

The test was run on mouse CTCF dataset (8 Mbp). Columns 3-5: 3) Times: running time under each word width K; 4) Motifs predicted: number of unique motifs found by each schema; and 5) E-value: the lowest e-value of alignments between a major motif found by each schema and motifs in mouse motif database.

#### Selecting the background model

The last parameter is the background model for motif finding. Background of the 1^st^-order Markov chain barely showed any difference with the 0^th^-order (i.e. without considering the dependency of neighbouring bases) in terms of detection ratio. Either the whole genome sequence or merely the target regions can be selected as the background model. During the accuracy assessment, we set the length of flanking region to be 650 bps. However, when it was set as 500 bps and 800 bps, the results were quite similar and robust.

#### Constructing a motif from overrepresented words

Clustering on primitive motifs was necessary for our methods. In the current version of MOST/MOST+, word instances were extended by substituting bases one by one. Therefore, only a limited space instead of the whole possible space was explored. For instance, in searching motif characterized by ATGCAAAT, it enumerated 4*8 = 32 single nucleotide substitutions to simulate position frequency matrices (PFM) whereas the actual possible solution space might be larger (for example, word instances with more than one substitutions, like A**CC**CAAAT, were not considered).

## CONCLUSION

Genome-wide epigenetic or DNase I signals can improve motif finding significantly. In this paper, we present MOST+, an accurate and fast motif discovering system through combining the genomic sequence and these genome-wide signals.

There are four highlights for MOST+: 1). It is one of the first *de novo *motif finding systems to combine the word frequency information and genome-wide signals from different sources such as histone modification information and DNase I hypersensitivity information. 2). It is a fast system for motif finding. MOST+ is 2 orders of magnitude faster than DREME. 3). It can deal with a large size of input data. For example, MOST+ can find motifs from an input data of 100 Mbps. 4). Its accuracy is at least comparable with the best existing systems.

## Methods

### Datasets

#### Mouse dataset

A benchmark ChIP-seq experiment of 13 mESC TFs associated with maintenance of pluripotency in mouse embryonic stem cell (cell type EF0_000462) [29] were collected. Nine epigenetic marks [22] in the same cell line were tested: DNase I, H3K27me3, H3K36me3, H3K4me1, H3K4me2, H3K4me3, H3K9me3, H3 and H4K20me3. Three of them (H3K4me1, H3K4me3, DNase I) were selected for subsequent analysis in which each TF against these three marks showed strong and distinct patterns of either kurtosis or single spike (Figure S7 and S8). We also included a raw ChIP-seq signal track. ChIP-seq peaks of each TF (controlled by removing peaks also found in anti-GFP ChIP-seq under the same cell line) are called using MACS [30]. All tracks were lifted and aligned to reference genome mm9.

For mESC date set, we included 146 motifs from JASPAR, 386 from UNIPROBE and 13 by Chen et al., 541 motifs in total, to form our mouse motifs database. We identified motifs using TOMTOM, a motif comparing and visualization tool [31]. Motif alignments with E-values less than 0.05 were considered statistically significant.

#### Human dataset

We also evaluated our method on human lymphoblastic cell line GM12878. 4 TFs were tested: CTCF, JUND, MAX (From UCSC genome browser) and VDR ChIP-seq (From Ramagopalan et al. [32]). DNase I hypersensitivity signals and histone modification marks H3k4me1, H3k4me2 and H3k4me3 (all from ENCODE project [33]) were included. ChIP-seq peaks were called by MACS. All tracks were mapped to human reference genome hg18. For human data we used 1,268 known motifs selected from JASPAR and UNIPROBE as the motif database (See table S3).

#### Genome-wide promoter regions of the mouse genome

An exhaustive search is conducted on the promoter regions of the whole mouse genome (upstream/downstream 1000bps of transcription start sites). The total size of the whole mouse promoter region is 89 Mbps. Transcription start site information (of mm9) was retrieved from UCSC RefSeq track. For time complexity comparison, different sizes of datasets are generated from this dataset by cutting this promoter region dataset.

### MOST+ pipelines

MOST+ contains two modes: one for word frequency counting only (MOST) and the other for combining different genome-wide signatures (MOST+). The general design of MOST+ is shown in Figure 1. MOST finds overrepresented words only from genomic sequences (such as a set of ChIP-seq peak regions of 200 bps), while MOST+ incorporates signals like histone modification marks and DNase I hypersensitivity sites to enhance the search of significant seeds. Finally, the program exports results including a list of found motifs and their corresponding position frequency matrices (PFM) (See Additional file 1 for more details).

### Exploiting epigenetic signals

Mark distributions nearby one particular site can indicate whether this site tends to be bound by TFs. However, it is weak and noisy. Thus we aggregated the aligned signals for all occurrences of a particular k-mer seed. We denote this overall distribution as mark distribution for this k-mer seed. The mark distribution is discretized over a set of fixed-size bins (illustrated by Figure S9).

### Finding over-represented seeds

MOST and MOST+ find each word's occurrences by utilizing a suffix tree. In order to reduce the space complexity, a hash table is utilized to represent the suffix tree. Ukkonen algorithm is employed to construct the suffix tree. Counting and locating each occurrence can be done by traversing the whole tree in asymptotically linear time (see Additional file 1 for more details).

The null model we adopted to find over-represented k-mer seeds was generated from an n^th^-order Markov chain [34] derived from the target regions. We assume the counts of each k-mer are in Poisson distribution. The p-values can be estimated using Gaussian approximation.

$$WC\sim Pois\left(\lambda \right)$$

(1) $$P\left(WC\geq wc\right)≅1-\phi \left(\frac{wc}{\lambda }\right),\lambda >10$$

Where wc and  λ denote the observed and simulated average counts of certain k-mer respectively.  ϕ is the cumulative distribution function of the standard normal distribution. For simplicity the calculation does not correct for the effects of self-overlapping words. We directly use word counts averaging on 5 simulated sequences with the same length of the query sequence, which was reported to have no substantial effect on result [14]. Nor do we correct for multiple testing, which is intractable since null odds ratio score distributions vary from word to word. Instead, we choose a small p-value threshold.

### Ranking words

MOST+ chooses a list of candidate motif seeds from all possible k-mers by applying a ranking step. If MOST+ mode is selected, aside from over-representation score, external experimental signals are decomposed into 3 sub-feature scores for each candidate seed: intensity scores (characterizing average signal intensity over all occurrence), kurtosis score (characterizing the peak pattern of a mark distribution) and asymmetry score (characterizing the irregularity of a mark distribution). To calculate kurtosis and asymmetry score the mark distribution was first normalized, then smoothed (by taking Discrete Fourier Transform and removing high frequency components). Finally we measured the kurtosis and asymmetry level. An overall score integrating all 4 scores above to rank candidate seeds is calculated as follows.

$$S=\beta_{I}S_{I}+\beta_{K}S_{K}+\beta_{A}S_{A}+S_{W}$$

$$S_{I}=\frac{1}{wc}\sum \varphi \left(X\right)$$

$$f\left(X\right)=smooth\left(normalize\left(\varphi \left(X\right)\right)\right)$$

$$S_{K}=\frac{E_{f}\left[{\left(X-EX\right)}^{4}\right]}{E_{f}{\left[{\left(X-EX\right)}^{2}\right]}^{2}}-3$$

(2) $$S_{A}=\frac{1}{2}D_{KL}(f_{lhs}||f_{rhs}\text{)}+\frac{1}{2}D_{KL}\left(f_{rhs}||f_{lhs}\right)$$

$$S_{W}=\frac{wc}{\lambda }$$

$$D_{KL}(p||q)=\underset{i}{\sum }p\left(i\right)\text{ln}\frac{p\left(i\right)}{q\left(i\right)}$$

Where *S_I _, S_K _,S_A _*and *S_w _*represent score of intensity, kurtosis, asymmetry and over-representing odds ratio. φ(X) is the aggregated distribution of the genome-wide signals. f(X) represents normalized and smoothed distribution. The  β values are weights for each score that need to be found. Symmetrised Kullback-Leibler divergence (KL-divergence) is adopted to measure the asymmetry of distributions. lhs and rhs denote left and right half of a distribution. woandλ are defined as in equation (1).

### Generalizing seeds to motifs and Clustering

For each high-ranked seed, we generalized them into motifs of the same length by substituting each position of a word with other 3 oligonucleotides, and calculated each variant's occurrence, which produced a probability matrix of this word.

In order to remove redundancy and form full-length motifs, these primary motifs were further clustered. Here we use a similar approach as CisFinder [17] which clusters words by PFMs since PFMs contain more site-specific details about each motif.

Our clustering procedure initially calculated a dissimilarity matrix according to primary motifs and corresponding mark distributions, which have a time complexity of *O(M^2^L^2^) *for *M *motifs with length *L*. All possible offsets and orientations of two motifs were checked to decide how they overlapped, and the one with maximum Pearson correlation was chosen as the distance of two motifs. If under MOST+ mode, the KL-divergence between mark distributions of these two motifs could be also incorporated to calculate this distance (Figure S10). Pseudo-counts could be added to avoid zero probability.

After that, an agglomerative hierarchical clustering was applied using average-linkage approaches (Figure S11). Gap statistics [35] was employed to determine the optimal cluster number. At last, clusters were trimmed to remove ambiguous or low-count ends.

### Assessment of site level accuracy

It is not straightforward to evaluate the accuracy of a motif discovery algorithm [2,8]. We evaluated the accuracy of our motif finding algorithm implicitly according to how well the found motifs can recall real sites.

With motifs found by MOST and other tested algorithms as inputs, we used CisFinder to predict bound sites on target regions. These predicted bound sites were compared with ChIP-seq signals as implicit evaluation of motif accuracy. However, as gold standard annotations, ChIP-seq has limited resolution. Therefore, we considered predicted sites that fell nearby ChIP-seq summits as true positives. Specifically, we flanked each ChIP-seq summit with 650bps at both sides (total 1,300bps for a single TFBS annotation, discretized into 13 100bp bins). The central bin of 100 bps was denoted as a TFBS bin (centred with the annotated TFBS summit), while other 12 bins at each end were denoted as flanking bins (Figure S12).

TP(TFBSbinshits#)=#ofsummitsthatarelessthan50bpfromanypredictedsites

$$FP\left(flanking\;bins\;hit\#\right)=\#\;of\;summits\;that\;are\;greater\;than\;50bp\;to\;all\;predicted\;sites$$

$$FN\left(TFBS\;bins\;missed\#\right)=\#\;of\;total\;TFBS\;bins-TP$$

TN(flankingbinsmissed#)=#oftotalflankingbins-FP

$$sensitivity=\frac{TP}{TP+FN},specificity=\frac{TN}{TN+FP}$$

We evaluated the performance with a nine-fold cross validation (Figure 4). We randomly divided all data into ten partitions with equal sizes. Eight training partitions were used for MOST+/MOST and other methods to *de novo *find motifs. Then these found motifs were used to pinpoint TFBS (using CisFinder) on the other two partitions - One validation partition for determining appropriate parameters, and the other partition for making comparison over algorithms. In case the essayed motif ranks lower in motif finding result compared with its cofactors, we consider the top 3 motifs for each TF dataset. Finally we compared the predictions with ChIP-seq annotation, and further drew ROC (Receiver Operator Characteristic) curve using above defined positives and negatives. AUROC (Area Under ROC) was derived and used as the measurement of accuracy. The pipeline was repeated on different assignment of training and validation partitions to calculate the mean and standard variation of AUROC.

### MOST+ parameter determination

During the training procedure, parameters of MOST+ need to be decided. MOST+ includes 4 parameters (intensity, kurtosis, asymmetry and clustering) concerning external tag information, 3 (intensity, kurtosis and asymmetry) during ranking phase and the remaining one in the clustering phase. To find sets of parameters for various TFs, the parameter spaces are searched systematically for a collection of discrete points, and the best parameter set is selected from thousands of combinations (See Additional file 1 for details).

### Comparison with existing methods

Existing methods, such as DREME, Trawler, WEEDER, HOMER2 and CisFinder, have been compared with MOST/MOST+. The command lines used to run these tools are listed at the end of the Additional file 1.

### Accessibility and requirement

MOST+ is implemented in C++. The source code is compiled with G++ 4.1.2. All tests were performed in a Linux server with CentOS 5.7, Intel Xeon E5520 @ 2.27GHz processors and a total memory of 16 Gigabytes. MOST+ and all supporting data are freely available at: http://cbb.sjtu.edu.cn/~ccwei/pub/software/MOST/MOST.php.

## Competing interests

The authors declare that they have no competing interests.

## Authors' contributions

CW and YZ conceived and designed the project; YZ implemented and tested the system; YZ, GZ and YH tested the system and analyzed the data; CW, YZ wrote the manuscript.

## Supplementary Material

Additional file 1 **This file contains supporting information for MOST+, including result figures S1 to S12, Tables S1 to S3 and some other detailed information like the parameter settings for the programs compared in this paper**. Click here for file

## Declarations

The publication charges for this article have been funded by the National Natural Science Foundation of China (61272250).

This article has been published as part of *BMC Genomics *Volume 16 Supplement 7, 2015: Selected articles from The International Conference on Intelligent Biology and Medicine (ICIBM) 2014: Genomics. The full contents of the supplement are available online at http://www.biomedcentral.com/bmcgenomics/supplements/16/S7.
