# Supplementary material for: MOST+: A de novo motif finding approach combining genomic sequence and heterogeneous genome-wide signatures
Source: BMC Genomics. 2015 Jun 11;16(Suppl 7):S13. doi: 10.1186/1471-2164-16-S7-S13 (PMC4474412; doi:10.1186/1471-2164-16-S7-S13)
Supplement: Additional file 1 — This file contains supporting information for MOST+, including result figures S1 to S12, Tables S1 to S3 and some other detailed information like the parameter settings for the programs compared in this paper. [file 1471-2164-16-S7-S13-S1.docx]

**SUPPLEMENTARY MATERIAL**

**MOST+: A de novo motif finding approach combining genomic sequence and heterogeneous genome-wide signatures**

**Yizhe Zhang, Yupeng He, Guangyong Zheng , Chaochun Wei**

**This file contains supporting information for MOST+, including figure S1 to S12, Table S1 to S3 and some other supporting information.**

**Supplementary Figure and Table Legends**

**Figures**

Figure S1. Joint motifs. Left panel: Oct4-N-Sox2 joint motif, tandem repeat for core OCT4 motif was found. Right panel: ESrrb alternative binding motif, including a palindrome.

Figure S2. Top 500 qualified motif seeds from ESrrb data set. Repeat words are associated with higher level of noise and asymmetry.

Figure S3. Comparison of CTCF binding motifs generated by different methods. From up to down, the methods are WEEDER (as in Chen et al.), CisFinder, DREME, MOST+ and MOST (Graphs were adapted from the outputs of TOMTOM).

Figure S4. Comparison of overall AUROC over 13 TFs.

Figure S5. Site level accuracy of MOST and MOST+ on human data.

Figure S6. Some known motifs found from promoter region (within 1000bps of transcriptional start site) . Upper panels are motif in database. Lower panels are motif found by MOST+

Figure S7. Two types of histone marks spatial distribution centered by ChIP-seq peaks, right panel for single spike(E2f1) and left panel for kurtosis (CTCF).

Figure S8. Distributions centered at ChIP-seq peaks for all 13 TFs in mESC data set.

Figure S9. External signals utilization schema. Signals are first mapped to the genome sequence to get their coordinates. Then sub-distributions around each occurrence are stacked together to help discover motifs.

Figure S10. Illustration for calculation on dissimilarity of motifs. Semantic distance and tag distribution distance are combined together by a weight parameter.

Figure S11. Hierarchical tree is made by dissimilar matrix. Representative of a cluster is chosen by minimizing the overall dissimilarity.

Figure S12. Schematic demonstration of positive and negative prediction.

**Tables**

Table S1. Running time (seconds) of each software.

Table S2 Optimal parameters for mouse data

Table S3. Data sources used in this work.


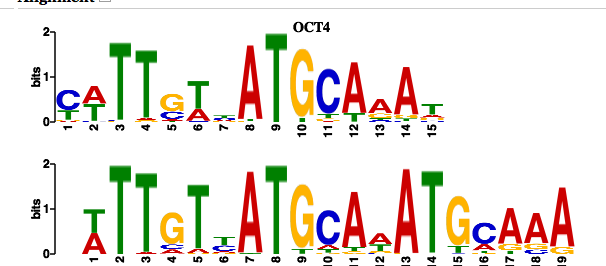


Figure S1. Joint motifs. Left panel: Oct4-N-Sox2 joint motif (up part) and tandem repeat for core OCT4 motif (below part). Right panel: ESrrb alternative binding motifs, including a palindrome.


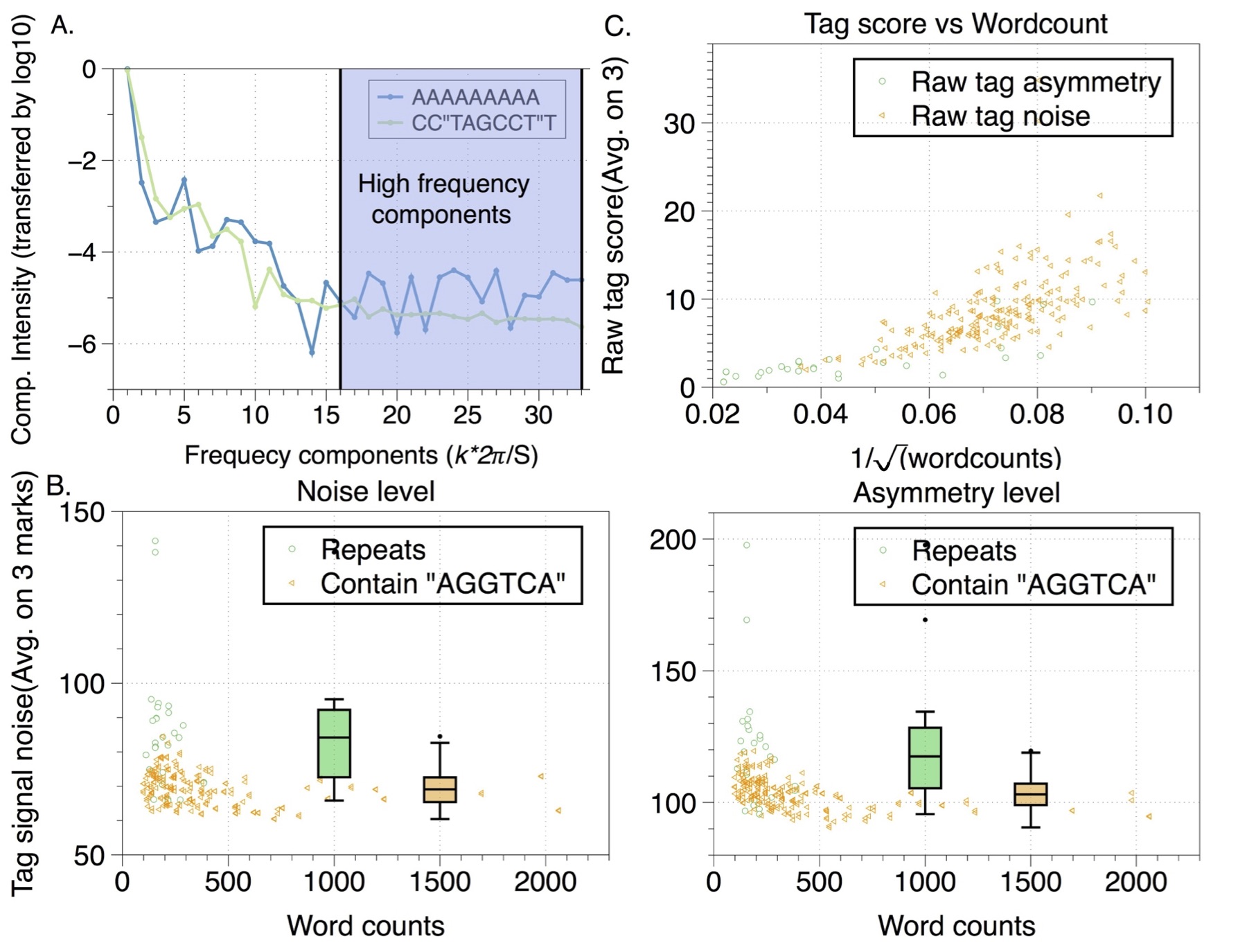


Figure S2. Top 500 qualified motif seeds from ESrrb data set. Repeat words are associated with higher levels of noise and asymmetry.

A: Words containing core feature of ESrrb (i.e. contain TAGCCT) show lower levels of high frequency noises.

B: Words containing core feature of ESrrb (i.e. contain TAGCCT) have lower scores in noise and asymmetry levels.

C: Raw tag noise and asymmetry scores are divided by the square root of word counts to remove their dependency on word counts)


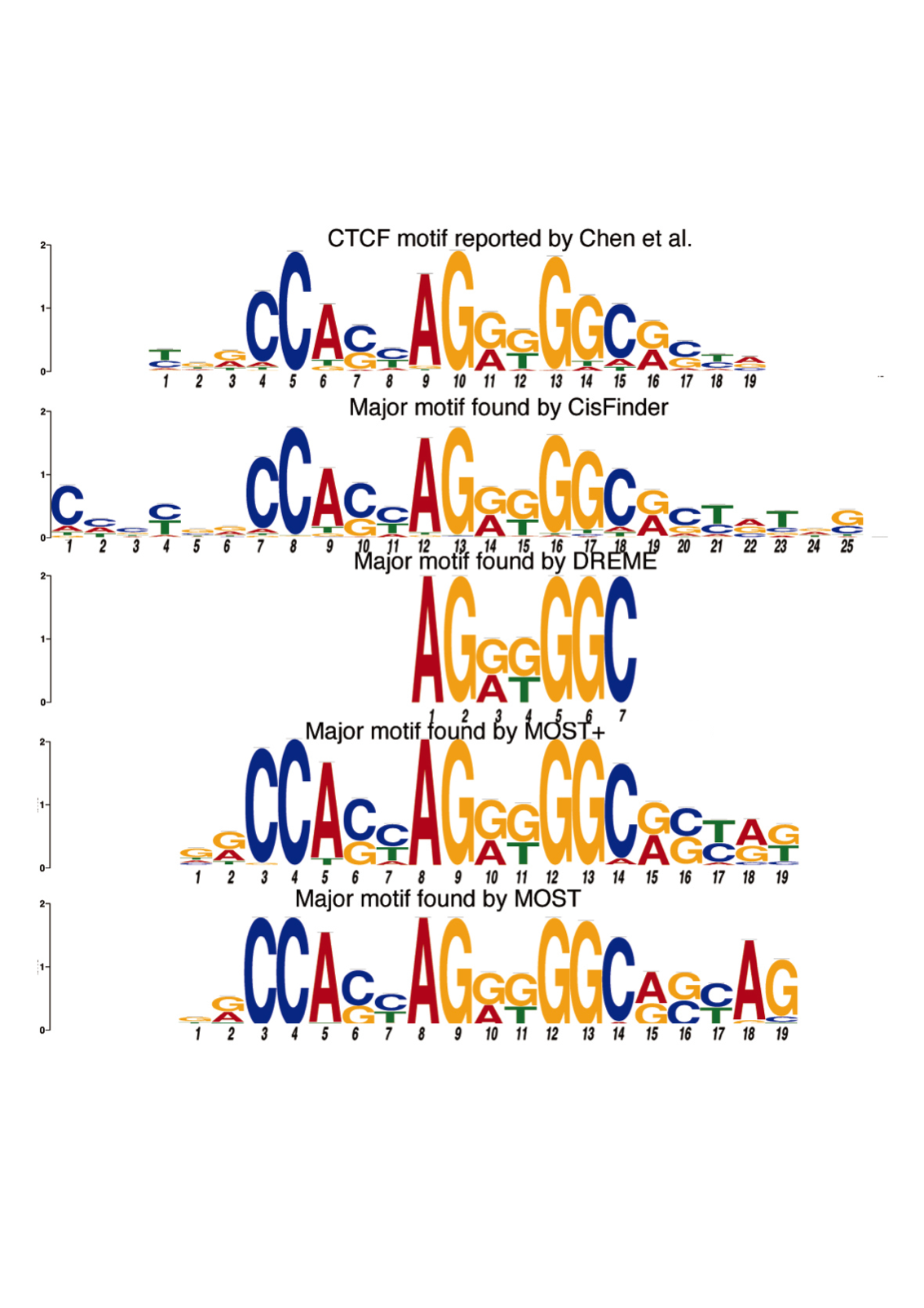


Figure S3. Comparison of CTCF binding motifs generated by different methods. From up to down, the methods are WEEDER (as in Chen et al.), CisFinder, DREME, MOST+ and MOST (Graphs were adapted from the outputs of TOMTOM).


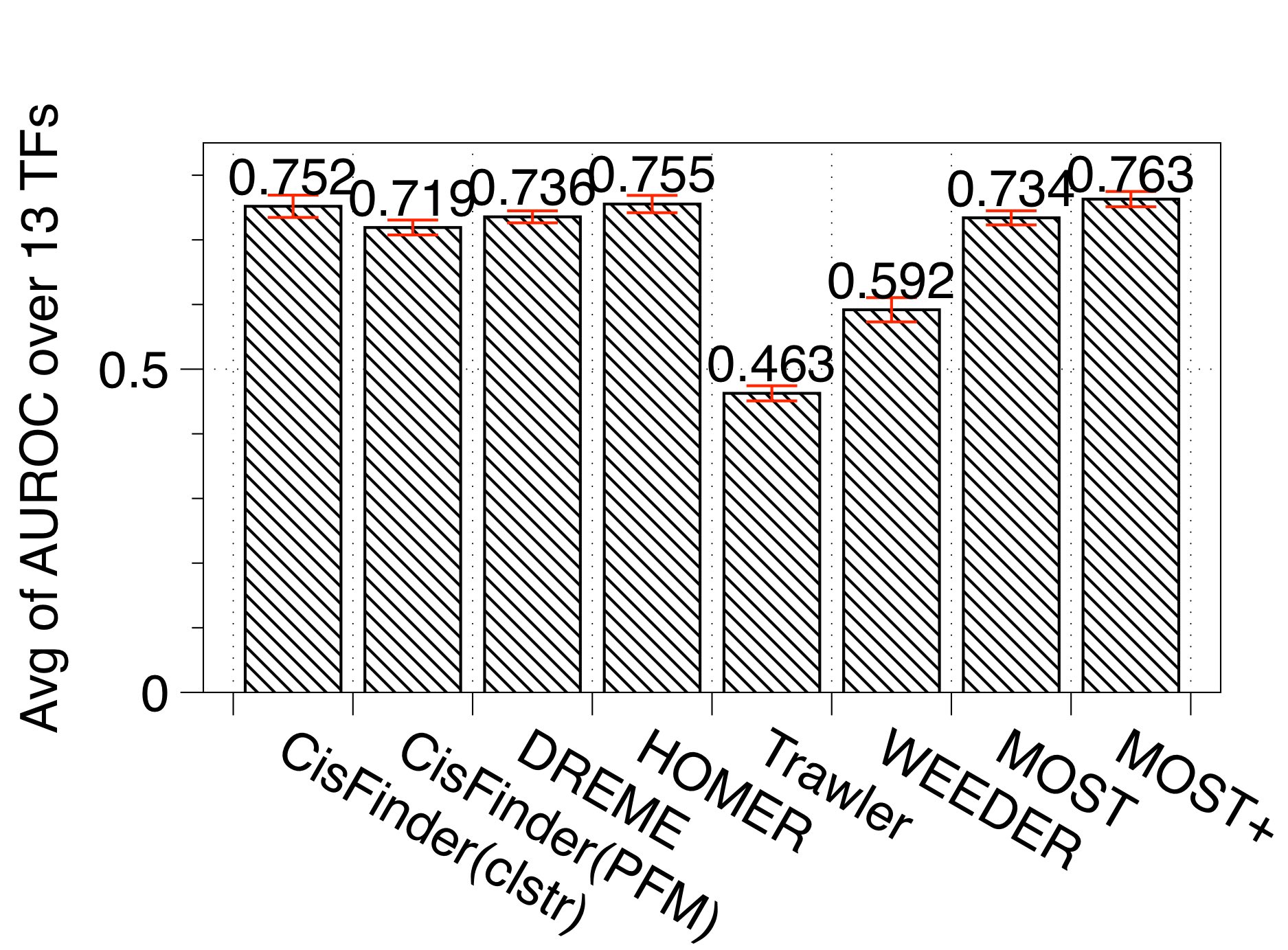


Figure S4. Comparison of overall AUROC over 13 TFs.


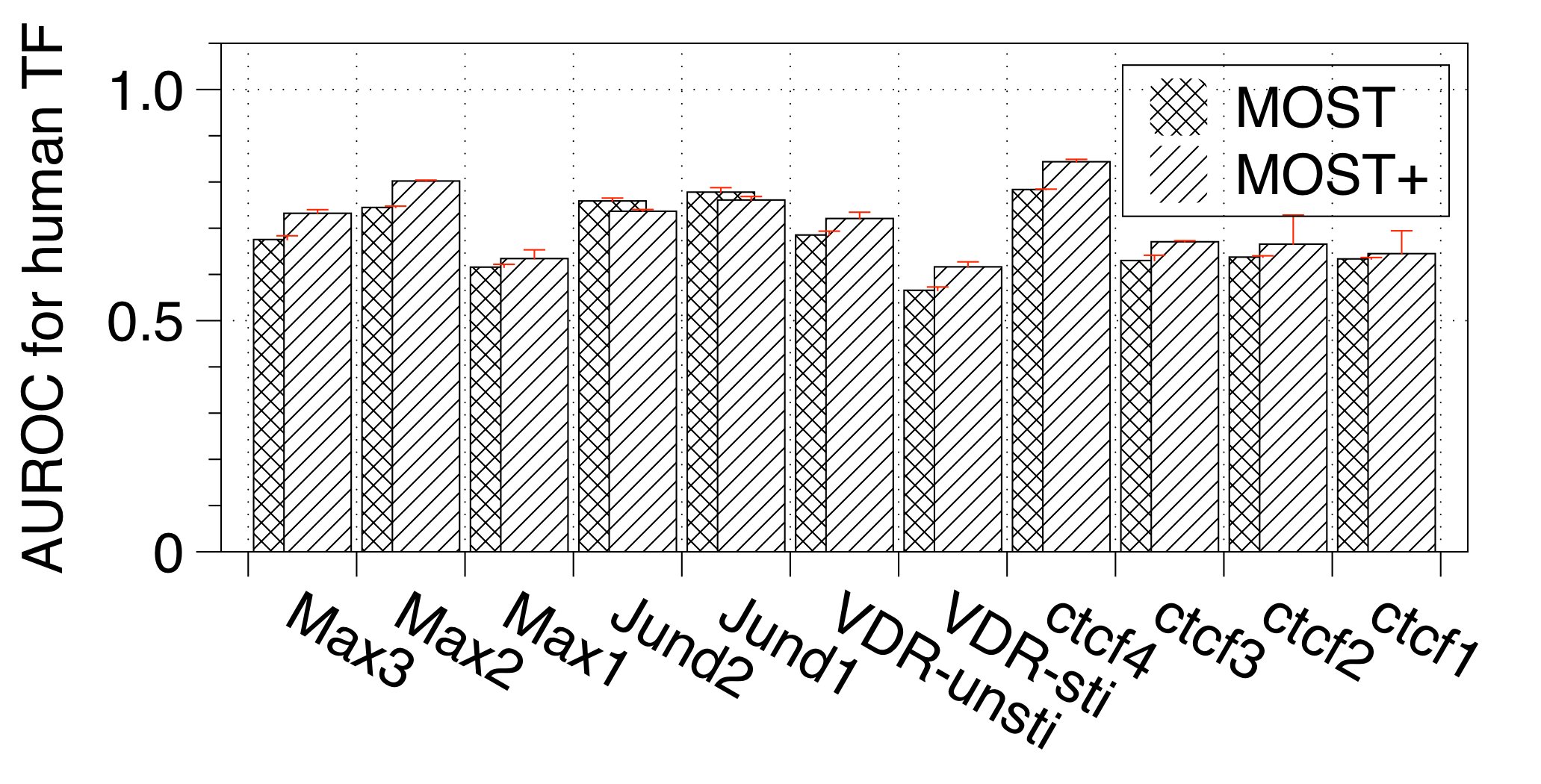


Figure S5. Site level accuracy of MOST and MOST+ on human data.

| GABPA | nfatc |
| --- | --- |
| 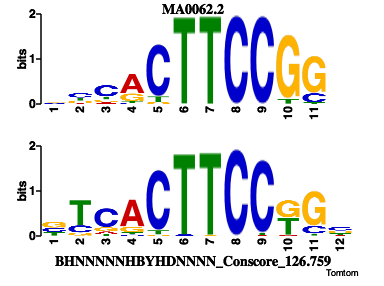 | 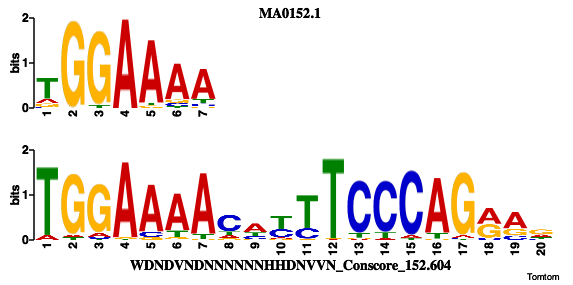 |
| Oct4 | creb(jundm2) |
| 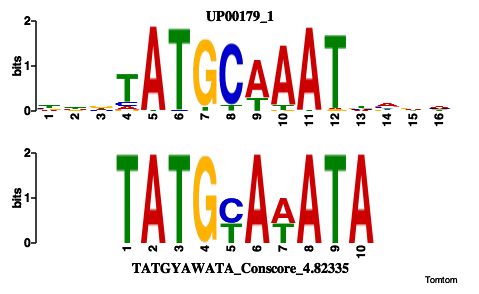 | 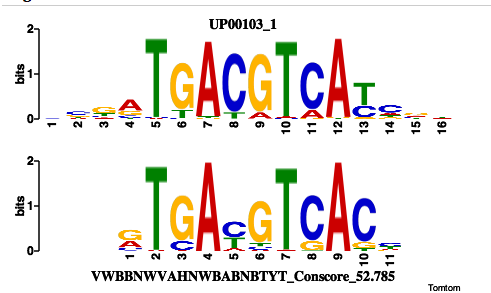 |
| NFYA | stat3 |
| 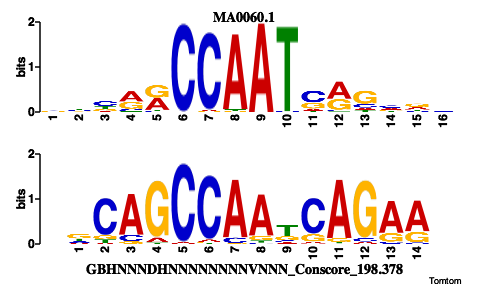 | 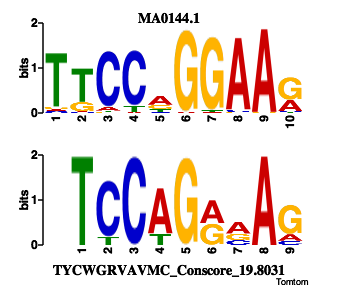 |

Figure S6. Some known motifs found from promoter region (within 1000bps of transcriptional start site). In each sub-figure, upper part is the motif in the database. Lower part is the motif found by MOST+.

Figure S7. Two types of histone marks signal distribution centered by ChIP-seq peaks, right panel for single spike(E2f1) and left panel for kurtosis (CTCF).

Figure S8. Distributions centered at ChIP-seq peaks for all 13 TFs in mESC data set.

Figure S8 (continued). Distributions centered at ChIP-seq peaks for all 13 TFs in mESC data set.

 **** Figure S8 (continued). Distributions centered at ChIP-seq peaks for all 13 TFs in mESC data set.

Figure S8 (continued). Distributions centered at ChIP-seq peaks for all 13 TFs in mESC data set.

Figure S9. External signals utilization schema. Signals are first mapped to the genome sequence to get their coordinates. Then sub-distributions around each occurrence are stacked together to help discover motifs.


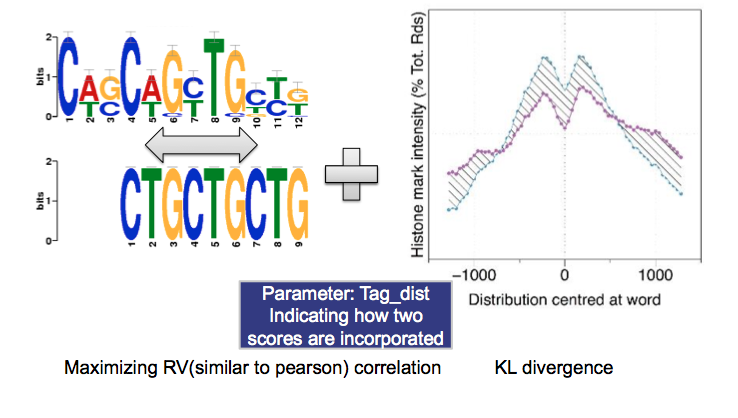


Figure S10. Illustration for calculation on dissimilarity of motifs. Semantic distance and tag distribution distance are combined together by a weight parameter.


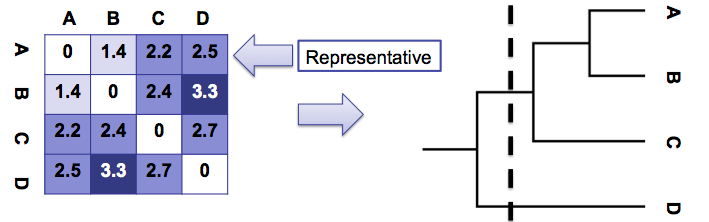


Figure S11. Hierarchical tree is made by dissimilar matrix. Representative of a cluster is chosen by minimizing the overall dissimilarity.


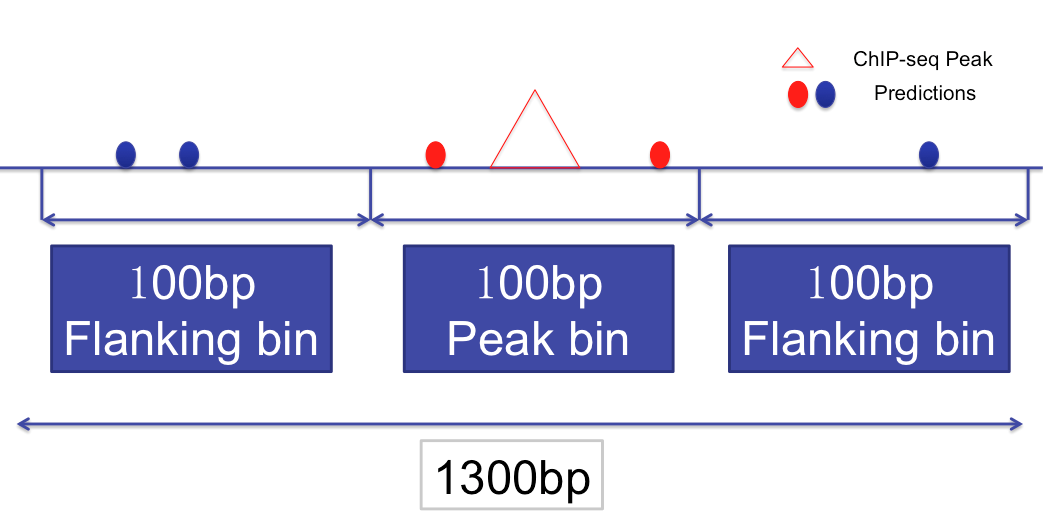


Figure S12. Schematic demonstration of positive and negative prediction.

**Tables**

Table S1. Running time (seconds) of each software.

| Sequnce size (Mbps) | 8 | 20 | 30 | 50 | 75 | 100 |
| --- | --- | --- | --- | --- | --- | --- |
| MOST(K=9) | 102 | 190 | 234 | 278 | 322 | 366 |
| MOST+(K=9) | 508 | 893 | 1034 | 1323 | 1634 | 1772 |
| DREME(K=7) | 8826 | 20868 | 36888 | 72909 | 118930 | 164951 |
| Trawler(mlength=6) | 741 | 11902 | 17483 | 23064.0 | 28644 | 34225 |
| WEEDER(w=6) | 42480 | 182521 | - | - | - | - |
| CisFinder | 153 | 253 | 480 | - | - | - |
| HOMER | 598 | 643 | 703 | 825 | 1039 | 1199 |

Table S2 Optimal parameters for mouse data (Bottom column: contribution to the improvement of AUROC, decided by whether this feature is included)

| Weights | Kurtosis | Asymmetry | Intensity | Clustering |
| --- | --- | --- | --- | --- |
| DNase I | 0.1 | 5 | 0 | 0.2 |
| H3K4me1 | 0 | 2 | 0.05 | 0 |
| H3K4me3 | 0 | 0.5 | 0.1 | 0 |
| TF ChIP-seq | 0 | 0 | 0.02 | 0 |
| Contribution | 1.3% | 4.3% | 1.1% | 0.7% |

Table S3. Data sources used in this work.

| ChIP-seq antibody or target protein | Source | Cell | Reference or lab |
| --- | --- | --- | --- |
| H3K4me1 (mouse) | GEO:GSE11172 | mESC | Meissner et al. |
| H3K4me3 (mouse) | GEO:GSE12241 | mESC | Mikkelsen et al. |
| DNase I (mouse) | DCC:EM003417 | mESC | Stam |
| Mouse 13 TFs | Publication | mESC | Chen et al. |
| VDR (Human) | Publication | GM12878 | Ramagopalan et al. |
| CTCF (Human) | ENCODEDCC | GM12878 | Bernstein |
| JUND (Human) | ENCODEDCC | GM12878 | Snyder |
| MAX (Human) | ENCODEDCC | GM12878 | Snyder |
| DNase I (Human) | ENCODEDCC | GM12878 | Stam |
| H3K4me1 (Human) | ENCODEDCC | GM12878 | Bernstein |
| H3K4me2 (Human) | ENCODEDCC | GM12878 | Bernstein |
| H3K4me3 (Human) | ENCODEDCC | GM12878 | Bernstein |

**Supplementary notes**

**Suffix Tree**

Ukkonen’s algorithm (1995) can reduce the time to build a suffix tree to O(n) time by using suffix link and other techniques. Then, our approach to determine the occurrence and loci for each K-mer (K-length word) can be described as 2 steps. First, we find a node in the tree that exactly represents or implicitly includes the K-mer we want to search. Next, we traverse the whole sub-tree of this K-mer to find the number of its leaf nodes and the total length of edge it has passed. If one exhaustively search every K-mer, it needs 4k edge-finding-steps (which can be done in a constant time if a hash table is adopted) to locate all of them, followed by less than 2N-4k steps to traverse sub-tree of each K-mer (where N is the length of query string, a suffix tree contains less than 2N-1 edges. In this case of quadtree, it approximately contains 4N/3 edges). Thus, in total it needs less than 2N steps to find occurrence and locations for each K-mer.

**Running MOST+ on different datasets**

3 types of tag signal files are supported: density file, wig with variant step sizes and wig with a fixed step size. Options can be made to output predicted loci in the genome, region sequences, tag distribution and clustering log file (containing each cluster’s member and alignment).

Check <http://cbb.sjtu.edu.cn/~ccwei/pub/software/MOST/MOST.php> for detailed instructions and latest MOST+ version.

For mESC and human LCL datasets, we use command lines as below (MOST+ ver.1.2):

Most –m tag –r <region.bed> -f <genome.fasta> -t <tags.wig>

Most –m normal –r <region.bed> -f <genome.fasta>

For promoter region, we use following commands:

Most –m tag –r <tss.bed> -f <genome.fasta> -t <tags.wig> -extend 1000 –cs 0.8 –rmrepeat 2

**Consensus contamination.**

Here we define consensus contamination: With misclassified words incorporated into cluster, the regular expression (shorted as RE, like CMCRCCC) of a cluster would be more ambiguous, rendering this cluster more vulnerable to other falsely classified words under our clustering setting. This could be proved that with help of histone marks, motifs found by MOST+ were better aligned to motifs databases.

**Parameter determination**

When we calculate the dissimilarity of two motifs, for each experimental signal we have 4 parameters. 3 parameters are for 3 sub-features (intensity, kurtosis, asymmetry), one for the mixing proportion of two distances in clustering step.

To find the optimal parameters for various TFs, we searched on parameter space by choosing a collection of discrete points (i.e. 0.05, 0.1, 0.2, 0.5, 1, 2, 5, 10) for each parameters. To simplify the search, we also assumed the optimal combination of parameters can be found by optimizing each parameter independently. The final optimal parameter combination only includes parameters that significantly improve the model (FDR q-value<0.05).

Despite of its roughness, the method is fair since each feature characterize a different aspect of a distribution. This approach dramatically reduces the computation cost. For the dataset we worked, we need 18,720 repeats on our pipeline, approximately 5 week CPU time on a typical server, to finish the whole test: 3Tags (Dnase, H3k4me1, H3k4me3) * 4 parameters (Noise, Symmetry, Bipeak, Intensity, cluster)*13 TFs*5 repeats * 8 values per parameter * first 3 motifs.

**Site-level accuracy tests**

Site-level accuracy has been compared for MOST and MOST+ in several scoring strategies in terms of ROC: Count Only (given by number of word instance around each position), Count-kurtosis joint score (given by adding word count number of each position with 1/10 of kurtosis score of the tag distribution centered at this position, charactering shape feature of a distribution) and Count-intensity joint score (given by adding word count number of each position with the total reads number around this position, characterizing intensity feature of a distribution). Sensitivity and specificity are estimated at site level in flanking regions (600bps upstream/downstream) of ChIP-seq peaks. When combined with tag kurtosis score, AUC of ROC increased, indicating shape feature may play an important role in recalling motif positions.

In site-level accuracy test, we use three different scoring strategies to give score for each position: one use genomic sequence only (Word-count-only strategy), other two combine word count and tag signal scores (count-kurtosis joint score and count-intensity joint score)

Word-count-only score is calculated by counting how many word instances can be found within 20bps around this position. For instance, if we use word width K=6 and a motif goes like ATCGATCGA, which is made up by 3 word member: ATCGAT, TCGATCG, GATCGA, sequence in a particular position is like …CTCGATCGAT…, then 2 word instances is found in this position: TCGATCG, GATCGA. Thus the score is set to 2. Since our algorithm focus on de novo motif discovery, this word-count-only is just a rough indicator of how well this position fit with motif.

Count-kurtosis joint score is given by adding count number of each position with 1/10 of kurtosis score of the tag distribution centered at this position (kurtosis score is defined in Methods session). This can be perceived as a justification on word-count-only score strategy by bring some additional tag distribution shape information into consideration.

Count-intensity joint score, a little bit different from Count-kurtosis joint score, it just add word counts with the total reads number around this position. Similar to what CENTIPEDE reported, this leads to a decrease in AUC under ROC.

**Optimal Parameter test**

Check <http://cbb.sjtu.edu.cn/~ccwei/pub/software/MOST/MOST.php> to download all parameter tests result.

**Running other algorithms and comparison tool (TOMTOM)**

We listed our command line with respect to each algorithm we tested, as below:

DREME:

dreme -eps -v 1 -p <seqfile> -e 0.05

MEME:

meme <seqfile> -dna -revcomp -nmotifs 20 -minw 4 -maxw 7

Trawler

trawler.pl -sample <seqfile> -background <negfile>

-directory <trawler out> -mlength 4

WEEDER:

weederlauncher.out <seqfile> <outfile> MM small S

HOMER2:

homer2 denovo -i <seqfile> -b <negfile>

CisFinder:

patternFind -i <seqfile> -o <pfwfile>

patternCluster -i <pfwfile> -o <clusterfile>

patternScan -i <clusterfile> -f <seqfile> -o <scanfile>

patternDistrib -i <scanfile> -f <freqfile> -a <andfile>

TOMTOM:

tomtom -no-ssc -min-overlap 5 -dist pearson -thresh 0.05 <motif.meme> <database>

For negative dataset we use dinucleotide shuffle program given in MEME Suite distribution:

Fasta-dinucleotide-shuffle –f <seqfile> -c 1 > <negfile>

For more information, check PPT and other files available in:

<http://cbb.sjtu.edu.cn/~ccwei/pub/software/MOST/MOST.php>
